# Dyspnea, Tachycardia, and New Onset Seizure as a Presentation of Wilms Tumor: A Case Report

**DOI:** 10.1155/2014/562672

**Published:** 2014-02-05

**Authors:** Linda Li, Jennifer Light, Michael Marchick, Robyn Hoelle

**Affiliations:** ^1^College of Medicine, University of Florida, Gainesville, FL 32610-0186, USA; ^2^Department of Emergency Medicine, University of Florida, 1329 SW 16th Street, P.O. Box 100186, Gainesville, FL 32610-0186, USA

## Abstract

Wilms tumor is found in 1 in 10,000 children and most commonly presents in asymptomatic toddlers whose care givers notice a nontender abdominal mass in the right upper quadrant. This case of Wilms tumor presented as a critically ill eleven-year old with significant tachypnea, dyspnea, vague abdominal pain, intermittent emesis, new onset seizure, metabolic acidosis, and hypoxemia. This is the first case in the literature of Wilms Tumor with cavoatrial involvement and seizure and pulmonary embolism resulting in aggressive resuscitation and treatment. Treatment included anticoagulation, chemotherapy, nephrectomy, and surgical resection of thrombi, followed by adjunctive chemotherapy with pulmonary radiation.

## 1. Introduction

Wilms tumor (WT) is found in 1 in 10,000 children. The classical diagnosis of WT is through the incidental finding of a palpable abdominal mass on an otherwise asymptomatic young child. This case describes an unusual presentation in an adolescent who presented with abdominal pain, emesis, shortness of breath, tachycardia with palpitations, and new onset seizure. The patient described presented to physicians in a critical state and appeared to have a cardiac or neurologically related diagnosis. This rare presentation of Wilms tumor should be regarded when abdominal pain patients present with a myriad of symptoms.

## 2. Case Report

An 11-year-old female presented to the emergency department after a week-long history of intermittent abdominal pain and was found to be tachycardic up to 190's. Six days prior to arrival she had her first episode of abdominal pain with emesis. She was at school and described the pain as vague and diffuse. The episode spontaneously resolved without intervention or medication, but she still complained of residual intermittent abdominal pain throughout the following week. Similar abdominal pain and nonbloody, nonbilious emesis brought her back to the school nurse on the day of ED visit. She had difficulty walking to the office due to dyspnea. The mother witnessed the patient having 2 episodes of seizure-like activity including clenching of the hands, stretching of the feet, and foaming at the mouth with lip cyanosis and incontinence lasting a total of 15 minutes.

Upon arrival at the ED, the patient was severely tachycardic with an ECG demonstrating sinus tachycardia at 190 bpm with intraventricular conduction delay. The tachycardia improved to 130 bpm after fluid resuscitation with normal saline. The patient recalled experiencing similar palpitations in the past week that worsened with activity and improved with rest. Patient and mother report never experiencing fainting or having a murmur and deny ever having a seizure, neurological deficit, or gait disturbance in the past.

An abdominal exam revealed slight right-sided distention that was tender with a palpable mass. Normoactive bowel sounds were present. Ear, nose and throat, respiratory, cardiovascular, musculoskeletal, lymphatic, skin, and gross neurologic exams after the postictal stage were all normal.

Abnormal lab results included elevated creatinine at 0.95 mg/dL, high lactate level of 7.94 mmol/L, and metabolic acidosis with respiratory compensation as indicated by a pH of 7.27 with reduced HCO^3−^ of 16.8 mmol/L and PCO_2_ of 38.2 mmHg. She also had anemia with a hemoglobin of 8.3 g/dL.

**Figure 1:**
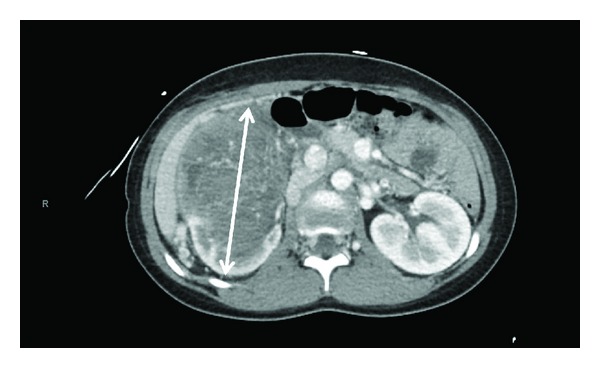
Intrarenal tumor of the right kidney.

An echocardiogram was performed due to the persistent tachycardia and showed the presence of a large echogenic, mobile polypoid, and pedunculated mass in the right atrium with attachment to the wall close to the IVC entry point that measures 6.8 × 2.8 cm during prolapse through the tricuspid valve. In addition to tumor thrombus detected within the right atrium, thrombi were also detected in both right and left pulmonary arteries by CT. The V/Q scan of the lungs revealed decreased perfusion involving the majority of the left lower lobe. Because of this finding and the seizure activity, a head CT and MR angiogram were performed and showed no evidence of acute intracranial abnormality. Stroke workup was unremarkable. A preliminary ultrasound of the abdomen revealed a large right renal mass with almost no normal renal parenchyma left. This finding prompted a CT scan. The CT of the abdomen showed a 13.2 × 10.6 × 16.5 cm intrarenal tumor of the right kidney (Figure 1), with possible rupture due to ill-defined margins anteriorly and medially. The tumor extended to the right renal hilus with invasion of the IVC. During inpatient admission, an ultrasound-guided biopsy of the right renal mass was performed, the result of which confirmed the diagnosis of Stage IV Wilms tumor with extension to the inferior vena cava, right heart, and lungs.

The patient underwent Heparin anticoagulation. Per protocol for Stage IV Wilms tumor, chemotherapy was initiated with Dactinomycin IV 2.3 mg once, and Vincristine IV 2 mg every seven days. After three rounds of chemotherapy, the patient responded positively to treatment with resolution of pulmonary metastatic disease. The primary right renal mass slightly decreased in size from 12.1 × 10.5 cm in axial dimensions to 10.3 × 8.5 cm. The patient then underwent a median sternotomy, with IVC and right atrial thrombus removal, and pulmonary endarterectomy in addition to a radical right nephrectomy on cardiopulmonary bypass.

She was monitored by video electroencephalography (EEG) throughout her hospitalization with no repeat seizure-like activity. No antiseizure medications had been given. Post-op, the patient developed a first-degree AV block that resolved within a week of surgery. On post-op day number 9, she is discharged to go home with prn Tylenol and oxycodone for pain management. Six months after her ED visit, she has had no repeat seizure-like activities or palpitations. The patient continued treatment with whole-abdominal radiation and chemotherapy.

## 3. Discussion 

WT is the most common childhood genitourinary tumor, affecting 1 in 10,000 children with an average age of diagnosis at 3 ys with the typical presentation of a caretaker noticing an abdominal mass in an otherwise well-appearing, healthy child [1]. Caval extension of WT is uncommon but not insignificant with a reported prevalence of 4–10% and intracardiac extension rarer at 1% [1–3]. A retrospective study identified 9 cases of intracaval and intracardiac involvement of WT that presented with abdominal pain and vomiting at an average age of 6.6 years [3]. In another review of 17 children with cavoatrial extension of WT, 71% presented with abdominal pain and no other symptoms of intravascular involvement [2]. In addition to the later age of diagnosis and abdominal pain presentation, this case also contains the rare occurrence of symptomatic intravascular involvement of WT as exemplified by the tachycardia, dyspnea, and seizure most likely from decreased cerebral perfusion.

It is uncommon for a WT patient to present with symptoms of cavoatrial involvement, and even more rare with seizure activity; there has been no prior documentation of seizure in the presentation of WT. In this case, the patient's experience of dyspnea, tachycardia, and seizure may be caused by the large tumor thrombus in the right atrium with extension to both left and right pulmonary arteries. One possible explanation is as follows. The patient may have developed hypoxemia with exertion that resulted in metabolic acidosis and other symptoms. The patient's tachypnea was respiratory compensation to the acidosis and was experienced as a significant shortness of breath. The heart attempted to increase cardiac output to improve oxygenation, causing tachycardia [4]. The tumor in the right atrium prolapsing through the tricuspid valve impeded ventricular filling. The pedunculating thrombus placed mechanical strain on the atrium during diastole and was potentially confounded by cardiac hypoxia leading to an arrhythmia, shown on the initial ECG as intraventricular conduction delay. The arrhythmia, in addition to the tachycardia, resulted in a transient reduction in cardiac output. In the absence of cerebral emboli, stroke, or other anatomical causes of seizures, as indicated by the normal CT and MR angiogram, the patient's newly onset seizure is likely due to transient cerebral hypoperfusion brought about by arrhythmia superimposed on tachycardia.

Although arrhythmia-induced seizures are rare, there has been an increase in their recognition and this patient had an accompanying hypoxia due to pulmonary thrombi [5, 6]. Schott et al. compiled 10 cases of cardiac arrhythmias that presented as activity identical to the tonic component of a typical epileptic seizure that stemmed from impaired cerebral perfusion due to a cardiac arrhythmia [6]. Though primary epilepsy is much more common, this patient's normal electrolyte and elevated glucose levels, afebrile state, and normal EEG indicate an arrhythmogenic seizure.

This patient's for stage IV WT with cavoatrial involvement consisted of anticoagulation, chemotherapy, surgical removal of tumor, and surgical resection of thrombi, followed by adjunctive chemotherapy with pulmonary radiation [7, 8]. Prognosis was determined by histology of the tumor. Four-year survival rate for this patient ranges between 73 and 75.3% compared to unfavorable histology of the same stage at 33.3% [7, 8].

This case presentation in an adolescent who presents with abdominal pain, seizure, tachycardia, and dyspnea is unprecedented. WT should not be disregarded as a diagnosis based on the age of the patient, but rather the clinician should also consider advanced disease in the context of multiorgan symptomatic presentation. A practicing physician should be aware of the possible variation of the clinical course and the resulting presentations of WT with metastases.

## Abbreviations

WT: Wilms tumor.
